# Neuropharmacological Exploration: Novel chalcone based small molecules ameliorate STZ induced memory dysfunction in mice for Cognitive Enhancement

**DOI:** 10.1002/alz70859_100144

**Published:** 2025-12-25

**Authors:** Manjinder Singh, Pratibha Sharma, Thakur Gurjeet Singh

**Affiliations:** ^1^ Chitkara College of Pharmacy, Chitkara University, Rajpura, Punjab India

## Abstract

**Background:**

One of the leading causes of dementia is Alzheimer’s disease, which initiated via plethora of biochemical alterations in brain of which Cholinergic, oxidative stress, and advance glycation end products (AGEs) have been identified as key mediators. Thus, the development of drugs targeting these pathways may help in managing AD.

**Method:**

Novel aminoalkylchalcone based molecules were designed and synthesized using aldol condensation method. In silico predication analysis was taken to determine the interactions of synthesized drugs with acetylcholinesterase. Memory enhancing effects were determined in‐vivo for most potent molecule against Streptozotocin induced memory deficit in mice. Possible mechanisms were studied by determining various brain biochemical parameters.

**Result:**

Novel aminoalkylchalcones were synthesized and showed strong interactions with crucial amino acids of target protein. Additionally, syntheses molecules showed strong AChE, DPPH and AGEs inhibitory activity determined in vitro. Furthermore, these novel compounds reversed STZ induced cognitive deficit (Morris water maze test) as well as oxidative stress, cholinergic hyperactivation and AGEs formation.

**Conclusion:**

Aminoalkylchalcones had potential to improve the cognitive functions via improving cholinergic transmission and reducing oxidative stress and AGEs. Thus, these potential molecules could be taken for further studies including toxicity evaluation to develop them as anti‐Alzheimer’s disease drugs.

